# Supplementary material for: Observation of the exceptional point in cavity magnon-polaritons
Source: Nat Commun. 2017 Nov 8;8:1368. doi: 10.1038/s41467-017-01634-w (PMC5676766; doi:10.1038/s41467-017-01634-w)
Supplement: Supplementary file 1 — Supplementary Information [file 41467_2017_1634_MOESM1_ESM.pdf]

Supplementary Note 1: Measurement setup

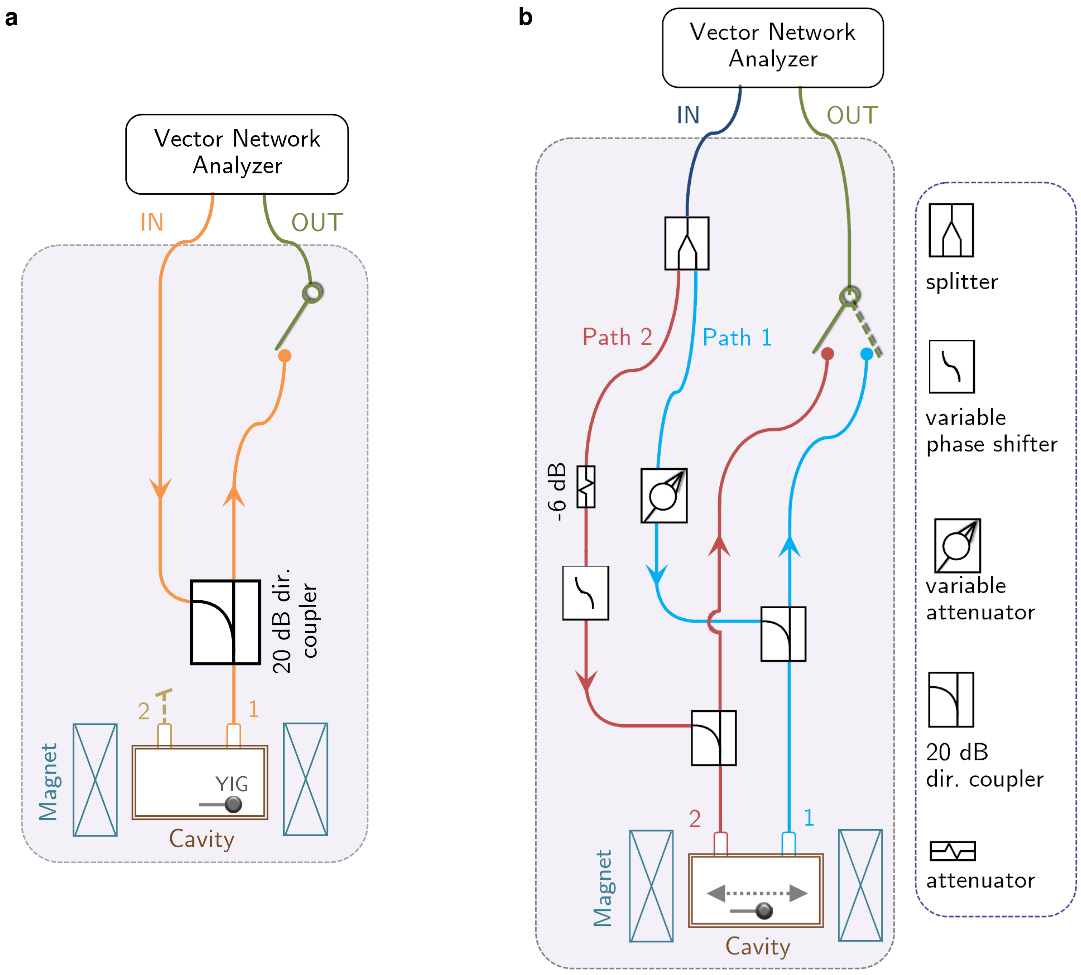

Supplementary Figure 1. **Measurement setup for the magnon-polariton system.** (**a**) Schematic diagram of the measurement setup with one feeding field. The microwave signal is injected into the cavity via port 1, reflected by the device, and detected using a vector network analyzer (VNA) via a 20 dB directional coupler. (**b**) Schematic diagram of the measurement setup with two feeding fields. The microwave signal generated by the VNA is divided into two copies and injected into the cavity via ports 1 and 2. The outputs from both ports are sent back to the VNA via two 20 dB directional couplers to measure the amplitude and phase responses of the device. In order to tune the power ratio and phase difference between the two feeding fields for ports 1 and 2, a variable attenuator and a phase shifter are placed in path 1 and path 2, respectively.

The measurement setup for a two-port cavity with a small YIG sphere embedded is schematically shown in Supplementary Figure 1. To evaluate the magnon-photon coupling strength and the damping rate of magnons, we first place the YIG sphere at the displacement $|x| = 11$ mm and measure its reflection spectrum (see Fig. 1e in the main text) with the measurement setup shown in Supplementary Figure 1a. Then, to observe polaritonic coherent perfect absorption (CPA), two feeding fields are injected into the cavity via ports 1 and 2, and the outputs from both ports are monitored (see Supplementary Figure 1b).

Supplementary Note 2: The Hamiltonian and spectra of the system

Effective non-Hermitian Hamiltonian of the system. According to the input-output theory[1], the quantum

Langevin equation for the annihilation operator a of a cavity mode is given, in the Markov approximation, by

$$\dot{a}(t) = -i\left[a(t), H_{\mathrm{s}}(t)\right] - \left(\kappa_{\mathrm{int}} + \sum_i \kappa_i\right) a(t) + \sqrt{2\kappa_{\mathrm{int}}}\, a_{\mathrm{int}}^{(\mathrm{in})}(t) + \sum_i \sqrt{2\kappa_i}\, a_i^{(\mathrm{in})}(t), \tag{1}$$

with

$$a(t) = e^{iHt} a e^{-iHt}, \quad H_{\mathrm{s}}(t) = e^{iHt} H_{\mathrm{s}} e^{-iHt}, \tag{2}$$

where the total Hamiltonian H includes the Hamiltonian H_{s} of the considered system, the Hamiltonian of the external field modes and the interaction Hamiltonian describing the couplings between them. In Supplementary Equation (1), κ_{int} is the intrinsic loss rate of the cavity mode, κ_i $(i = 1, 2)$ is the decay rate of the cavity mode due to the ith port, and $a_{\mathrm{int}}^{(\mathrm{in})}(t)$ and $a_i^{(\mathrm{in})}(t)$ are input fields related to the decay rates κ_{int} and κ_i. In our experiment, both κ_1 and κ_2 can be tuned by adjusting the pin lengths of ports 1 and 2 inside the cavity. Similarly, $a(t)$ can be related to the output fields $a_{\mathrm{int}}^{(\mathrm{out})}(t)$ and $a_i^{(\mathrm{out})}(t)$ as

$$\dot{a}(t) = -i\left[a(t), H_{\mathrm{s}}(t)\right] + \left(\kappa_{\mathrm{int}} + \sum_i \kappa_i\right) a(t) - \sqrt{2\kappa_{\mathrm{int}}}\, a_{\mathrm{int}}^{(\mathrm{out})}(t) - \sum_i \sqrt{2\kappa_i}\, a_i^{(\mathrm{out})}(t). \tag{3}$$

Subtraction of Supplementary Equation (3) from Supplementary Equation (1) gives

$$\sqrt{2\kappa_{\mathrm{int}}}\left[a_{\mathrm{int}}^{(\mathrm{out})}(t) + a_{\mathrm{int}}^{(\mathrm{in})}(t)\right] + \sum_i \sqrt{2\kappa_i}\left[a_i^{(\mathrm{out})}(t) + a_i^{(\mathrm{in})}(t)\right] = 2\left(\kappa_{\mathrm{int}} + \sum_i \kappa_i\right) a(t). \tag{4}$$

Because the above equation is obeyed for arbitrary values of κ_{int} and κ_i, it follows that

$$a_{\mathrm{int}}^{(\mathrm{out})}(t) + a_{\mathrm{int}}^{(\mathrm{in})}(t) = \sqrt{2\kappa_{\mathrm{int}}}\, a(t), \tag{5}$$

$$a_i^{(\mathrm{out})}(t) + a_i^{(\mathrm{in})}(t) = \sqrt{2\kappa_i}\, a(t) \quad (i = 1, 2), \tag{6}$$

which relate both input and output fields to the intra-cavity field.

Usually, there is no input field related to the intrinsic loss of the intra-cavity field. Thus, $a_{\mathrm{int}}^{(\mathrm{in})}(t) = 0$, and Supplementary Equation (1) is reduced to

$$\dot{a}(t) = -i\left[a(t), H_{\mathrm{s}}(t)\right] - \left(\kappa_{\mathrm{int}} + \sum_i \kappa_i\right) a(t) + \sum_i \sqrt{2\kappa_i}\, a_i^{(\mathrm{in})}(t). \tag{7}$$

Here we consider the perfect field-feeding case in which the incident fields injected into the cavity via ports 1 and 2 have zero output, i.e., $a_1^{(\mathrm{out})}(t) = 0$ and $a_2^{(\mathrm{out})}(t) = 0$. Then, from Supplementary Equation (6), it follows that

$$a_1^{(\mathrm{in})}(t) = \sqrt{2\kappa_1}\, a(t), \; a_2^{(\mathrm{in})}(t) = \sqrt{2\kappa_2}\, a(t). \tag{8}$$

Substituting Supplementary Equation (8) into Supplementary Equation (7), we obtain

$$\dot{a}(t) = -i\left[a(t), H_{\mathrm{s}}(t)\right] + \left(\kappa_1 + \kappa_2 - \kappa_{\mathrm{int}}\right) a(t). \tag{9}$$

For low-lying excitations of spin waves, the Hamiltonian of the cavity-magnon system is given by[2–4]

$$H_{\mathrm{s}} = \omega_{\mathrm{c}} a^\dagger a + \omega_{\mathrm{m}} b^\dagger b + g_{\mathrm{m}}\left(a b^\dagger + a^\dagger b\right), \tag{10}$$

where ω_{m} is the angular frequency of the magnon mode (here we only consider the Kittel mode) and g_{m} is the coupling strength between the photons in the cavity and the Kittel-mode magnons in the YIG sphere. From Supplementary Equation (10), it can be derived that

$$[a, H_{\mathrm{s}}] = \omega_{\mathrm{c}} a + g_{\mathrm{m}} b. \tag{11}$$

Substituting it into Supplementary Equation (9), we have

$$\begin{aligned} \dot{a}(t) &= -i\omega_{\mathrm{c}} a(t) - i g_{\mathrm{m}} b(t) + \left(\kappa_1 + \kappa_2 - \kappa_{\mathrm{int}}\right) a(t) \\ &= -i\left[\omega_{\mathrm{c}} + i\left(\kappa_1 + \kappa_2 - \kappa_{\mathrm{int}}\right)\right] a(t) - i g_{\mathrm{m}} b(t). \end{aligned} \tag{12}$$

Similarly, according to the input-output theory, the quantum Langevin equation for the annihilation operator b of a magnon mode is given, in the Markov approximation, by

$$\begin{aligned}\dot{b}(t) &= -i\left[b(t), H_{\mathrm{s}}(t)\right] - \gamma_{\mathrm{m}} b(t) + \sqrt{2\gamma_{\mathrm{m}}}\, b^{(\mathrm{in})}(t) \\ &= -i\omega_{\mathrm{m}} b(t) - i g_{\mathrm{m}} a(t) - \gamma_{\mathrm{m}} b(t) + \sqrt{2\gamma_{\mathrm{m}}}\, b^{(\mathrm{in})}(t).\end{aligned} \tag{13}$$

For the intrinsic damping rate γ_{m} of the magnon mode, there is no input field related to it, so $b^{(\mathrm{in})}(t) = 0$. Thus, Supplementary Equation (13) is reduced to

$$\dot{b}(t) = -i\left(\omega_{\mathrm{m}} - i\gamma_{\mathrm{m}}\right) b(t) - i g_{\mathrm{m}} a(t). \tag{14}$$

The equations of motion in Supplementary Equations (12) and (14) can be rewritten as

$$\dot{a}(t) = -i\left[a(t), H_{\mathrm{eff}}\right], \quad \dot{b}(t) = -i\left[b(t), H_{\mathrm{eff}}\right], \tag{15}$$

where

$$H_{\mathrm{eff}} = \left[\omega_{\mathrm{c}} + i\left(\kappa_1 + \kappa_2 - \kappa_{\mathrm{int}}\right)\right] a^\dagger a + \left(\omega_{\mathrm{m}} - i\gamma_{\mathrm{m}}\right) b^\dagger b + g_{\mathrm{m}}\left(a b^\dagger + a^\dagger b\right) \tag{16}$$

is the effective non-Hermitian Hamiltonian of the cavity-magnon system. Note that here we consider the perfect field-feeding case, which corresponds to the coherent perfect absorption (CPA)[5]. Furthermore, ω_{m} is tuned in resonance with ω_{c} (i.e., $\omega_{\mathrm{c}} = \omega_{\mathrm{m}} = \omega_0$) and also the relation $\kappa_1 + \kappa_2 - \kappa_{\mathrm{int}} = \gamma_{\mathrm{m}}$ is satisfied by tuning the system parameters, so the effective non-Hermitian Hamiltonian is reduced to

$$H_{\mathrm{eff}} = \left(\omega_0 + i\gamma_{\mathrm{m}}\right) a^\dagger a + \left(\omega_0 - i\gamma_{\mathrm{m}}\right) b^\dagger b + g_{\mathrm{m}}\left(a b^\dagger + a^\dagger b\right). \tag{17}$$

It can be found that the Hamiltonian in Supplementary Equation (17) satisfies $[PT, H_{\mathrm{eff}}] = 0$. Thus, the system is effectively described by a non-Hermitian PT-symmetric Hamiltonian[6,7]. The corresponding eigenvalues can be solved as

$$\omega_{1,2} = \omega_0 \pm \sqrt{g_{\mathrm{m}}^2 - \gamma_{\mathrm{m}}^2}. \tag{18}$$

In Supplementary Equation (18), the two eigenfrequencies $\omega_{1,2}$ are functions of ω_0, γ_{m} and g_{m}. To have a real spectrum, it requires that $g_{\mathrm{m}} > \gamma_{\mathrm{m}}$. For $g_{\mathrm{m}} = \gamma_{\mathrm{m}}$ in particular, $\omega_{1,2}$ coalesce to the central frequency ω_0. However, when $g_{\mathrm{m}} < \gamma_{\mathrm{m}}$, $\omega_{1,2}$ become complex and the PT symmetry is spontaneously broken. The spontaneous PT-symmetric breaking point at $g_{\mathrm{m}} = \gamma_{\mathrm{m}}$ is also referred to as the exceptional point.

Polaritonic CPA conditions. For the two-port cavity with a YIG sphere embedded, the transmission and reflection coefficients can be derived as

$$S_{21}(\omega) = S_{12}(\omega) = -\frac{2\sqrt{\kappa_1\kappa_2}}{i\left(\omega - \omega_{\mathrm{c}}\right) - \left(\kappa_1 + \kappa_2 + \kappa_{\mathrm{int}}\right) + \frac{g_{\mathrm{m}}^2}{i(\omega - \omega_{\mathrm{m}}) - \gamma_{\mathrm{m}}}}, \tag{19}$$

$$S_{11}(\omega) = -1 - \frac{2\kappa_1}{i\left(\omega - \omega_{\mathrm{c}}\right) - \left(\kappa_1 + \kappa_2 + \kappa_{\mathrm{int}}\right) + \frac{g_{\mathrm{m}}^2}{i(\omega - \omega_{\mathrm{m}}) - \gamma_{\mathrm{m}}}}, \tag{20}$$

$$S_{22}(\omega) = -1 - \frac{2\kappa_2}{i\left(\omega - \omega_{\mathrm{c}}\right) - \left(\kappa_1 + \kappa_2 + \kappa_{\mathrm{int}}\right) + \frac{g_{\mathrm{m}}^2}{i(\omega - \omega_{\mathrm{m}}) - \gamma_{\mathrm{m}}}}. \tag{21}$$

To achieve CPA in this cavity-magnon system, besides $\omega_{\mathrm{c}} = \omega_{\mathrm{m}} = \omega_0$ and $\kappa_1 + \kappa_2 - \kappa_{\mathrm{int}} = \gamma_{\mathrm{m}}$, there is also a constraint on the two feeding fields. We assumed that the two feeding fields have phase difference $\Delta\phi$ and power ratio q. The outgoing fields of ports 1 and 2 can be written as

$$S_1^- = \sqrt{q} e^{-j\Delta\phi} S_{11} + S_{12}, \tag{22}$$

$$S_2^- = S_{22} + \sqrt{q} e^{-j\Delta\phi} S_{21}. \tag{23}$$

The CPA requires zero output of the two ports, i.e.,

$$\left|S_{\mathrm{tot}}^-\right|^2 = \left|S_1^-\right|^2 + \left|S_2^-\right|^2 = 0. \tag{24}$$

Combining $\omega_c = \omega_m = \omega_0$, $\kappa_1 + \kappa_2 - \kappa_{int} = \gamma_m$, and Supplementary Equations (19–24), we obtain $\Delta\phi = 0$ and $q = \kappa_1/\kappa_2$. More explicitly, let us see the zeros of $\left|S_{tot}^{-}\right|^2$ under these specific conditions. First, Supplementary Equations (19–21) can be reduced to

$$S_{21}(\omega) = S_{12}(\omega) = -\frac{2\sqrt{\kappa_1\kappa_2}}{Q - 2\kappa_{int} + g_m^2/Q}, \tag{25}$$

$$S_{11}(\omega) = -1 - \frac{2\kappa_1}{Q - 2\kappa_{int} + g_m^2/Q}, \tag{26}$$

$$S_{22}(\omega) = -1 - \frac{2\kappa_2}{Q - 2\kappa_{int} + g_m^2/Q}, \tag{27}$$

where $Q = i(\omega - \omega_0) - \gamma_m$. Then, Supplementary Equations (22) and (23) are rewritten as

$$S_1^- = \frac{\sqrt{q}(|Q|^2 - g_m^2)}{Q^2 - 2Q\kappa_{int} + g_m^2}, \tag{28}$$

$$S_2^- = \frac{|Q|^2 - g_m^2}{Q^2 - 2Q\kappa_{int} + g_m^2}. \tag{29}$$

From Supplementary Equations (28) and (29), it can be verified that the two zeros of Supplementary Equation (24) exactly correspond to $\omega_{1,2}$ in Supplementary Equation (18).

Supplementary Note 3: Cavity transmission modified by the YIG sphere

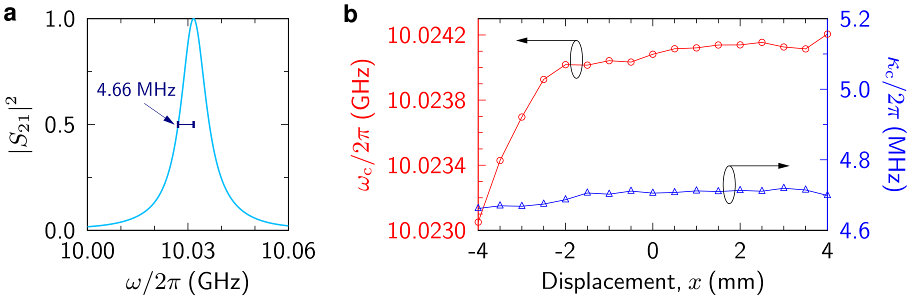

Supplementary Figure 2. Central frequency and linewidth of the cavity mode. (**a**) Transmission spectrum $|S_{21}|^2$ of the bare cavity. (**b**) Central frequency ω_c (red open-circle curve) and linewidth κ_c (blue open-triangle curve) of the cavity mode versus the displacement of the YIG sphere.

In the main text, we have discussed and measured the decay rates for the bare cavity (see Fig. 2a in the main text). To perform the experiment of CPA, $\kappa_1/2\pi$ and $\kappa_2/2\pi$ are tuned to be 1.72 and 1.39 MHz, respectively, for bare cavity. The transmission spectrum of the bare cavity is displayed in Supplementary Figure 2a, where $\omega_c/2\pi = 10.0317$ GHz and $\kappa_c/2\pi = 4.66$ MHz. However, when the YIG sphere is inserted into the cavity, both the central frequency and the total decay rate of the cavity mode are slightly changed for different displacements of the YIG sphere. To consider this effect in the analyzes and simulations, the central frequency and the cavity-mode linewidth versus the displacement of the YIG sphere are measured and plotted in Supplementary Figure 2b. It can be seen that $\omega_c/2\pi$ varies in the range of 10.0230–10.0242 GHz and $\kappa_c/2\pi$ in the range of 4.66–4.72 MHz when the YIG sphere is placed in the cavity.

With the measured values of $\kappa_1/2\pi$, $\kappa_2/2\pi$ and $\kappa_c/2\pi$, the intrinsic loss rate of the bare cavity is obtained as $\kappa_{int}/2\pi = 1.55$ MHz. Note that the port-induced decay rates κ_1 and κ_2 remain nearly unchanged when the small YIG sphere is embedded in the cavity, but the presence of the YIG sphere at different inner places of the cavity modifies the intrinsic loss rate κ_{int} of the cavity. From the values of $\kappa_1/2\pi$ and $\kappa_2/2\pi$ as well as the values of $\kappa_c/2\pi$ given in Supplementary Figure 2b, we obtain that the intrinsic loss rate $\kappa_{int}/2\pi$ of the cavity varies in the range of 1.55–1.61 MHz when the YIG sphere moves along the long edge of the cavity.

Supplementary References

[1] Walls, D. F. & Milburn, G. J. *Quantum Optics* 2nd edn. (Springer-Verlag Berlin Heidelberg, 2008).

[2] Tabuchi, Y. *et al.* Hybridizing ferromagnetic magnons and microwave photons in the quantum limit. *Phys. Rev. Lett.* **113**, 083603 (2014).

[3] Zhang, D. *et al.* Cavity quantum electrodynamics with ferromagnetic magnons in a small yttrium-iron-garnet sphere. *npj Quantum Inf.* **1**, 15014 (2015).

[4] Zhang, X., Zou, C.-L., Jiang, L. & Tang, H. X. Strongly coupled magnons and cavity microwave photons. *Phys. Rev. Lett.* **113**, 156401 (2014).

[5] Zanotto, S. *et al.* Perfect energy-feeding into strongly coupled systems and interferometric control of polariton absorption. *Nat. Phys.* **10**, 830–834 (2014).

[6] Heiss, W. D. The physics of exceptional points. *J. Phys. A: Math. Theor.* **45**, 444016 (2012).

[7] Kang, M., Liu, F. & Li, J. Effective spontaneous $\mathcal{PT}$-symmetry breaking in hybridized metamaterials. *Phys. Rev. A* **87**, 053824 (2013).
